# Optical ultrasound sensors for photoacoustic imaging: a review

**DOI:** 10.1117/1.JBO.29.S1.S11523

**Published:** 2024-02-01

**Authors:** Liying Zhu, Hongming Cao, Jun Ma, Lidai Wang

**Affiliations:** a City University of Hong Kong, Department of Biomedical Engineering, Kowloon, Hong Kong, China; b Nanfang Hospital, Southern Medical University, Department of Burns, Guangzhou, China

**Keywords:** photoacoustic imaging, ultrasound detection, all-optical detection, fiber sensor

## Abstract

**Significance:**

Photoacoustic (PA) imaging is an emerging biomedical imaging modality that can map optical absorption contrast in biological tissues by detecting ultrasound signal. Piezoelectric transducers are commonly used in PA imaging to detect the ultrasound signals. However, piezoelectric transducers suffer from low sensitivity when the dimensions are reduced and are easily influenced by electromagnetic interference. To avoid these limitations, various optical ultrasound sensors have been developed and shown their great potential in PA imaging.

**Aim:**

Our study aims to summarize recent progress in optical ultrasound sensor technologies and their applications in PA imaging.

**Approach:**

The commonly used optical ultrasound sensing techniques and their applications in PA systems are reviewed. The technical advances of different optical ultrasound sensors are summarized.

**Results:**

Optical ultrasound sensors can provide wide bandwidth and improved sensitivity with miniatured size, which enables their applications in PA imaging.

**Conclusions:**

The optical ultrasound sensors are promising transducers in PA imaging to provide higher-resolution images and can be used in new applications with their unique advantages.

## 1 Introduction

Photoacoustic (PA) imaging is a rapidly developing imaging technology that melds the high contrast of optical imaging with the deep penetration of ultrasound imaging.^1^ The principle of PA imaging is based on PA effect, which means the sample can generate ultrasound waves after absorbing light due to the thermoelastic expansion.^2^ By capturing these light-induced ultrasound waves, which focus deeper than light in most biological tissues due to reduced scattering, PA imaging delivers high-resolution, high-contrast images. Furthermore, with multiwavelength detection and computation, PA imaging offers functional information.^3^^–^^6^ PA imaging has been developed for more biomedical fields, such as oncology,^7^^–^^9^ cardiology,^10^^–^^12^ and ophthalmology.^13^^–^^15^

According to the different image formation methods, there are two major implementations of PA imaging: photoacoustic computed tomography (PACT) and photoacoustic microscopy (PAM).^16^^,^^17^ PACT employs an ultrasonic transducer array to capture PA signals from a sample illuminated by a broad optical beam, with images reconstructed using specific algorithms. In contrast, PAM typically employs a point-by-point scanning mechanism for image acquisition. Upon each pulsed laser excitation, the ultrasonic transducer of a PAM system collects the time-resolved PA signal. Based on the focusing configuration, PAM is subdivided into optical-resolution PAM (OR-PAM) and acoustic-resolution PAM (AR-PAM). In OR-PAM, the PA signal originates from the optical focal zone, with its lateral resolution governed by the diffraction-limited optical focus size. Meanwhile, AR-PAM captures the PA signal from a quasifocused light beam using a focused ultrasonic transducer, and its lateral resolution is determined by the acoustic focal spot size. For both OR-PAM and AR-PAM, the axial resolution predominantly depends on the ultrasonic transducer’s bandwidth.^18^ PA endoscopy (PAE) is essentially a variant of PAM, grounded in the same principles. However, it typically incorporates a compact probe and captures PA images through rotational scanning^19^ or MEMS mirror scanning.^20^

The ultrasonic transducer plays a pivotal role in PA imaging, influencing the system’s sensitivity, resolution, and size.^21^^,^^22^ There are three main types of ultrasonic transducers used in PA imaging, including piezoelectric transducers, micromachined ultrasonic transducers (MUTs), and optical ultrasound sensors.^23^ Piezoelectric transducers are made of piezomaterials, such as single crystals, piezoceramics, and polyvinylidene difluoride. Currently, the piezoelectric transducer is the most commonly used one in PA imaging due to its high sensitivity, high stability, and low cost. To further improve the sensitivity, bandwidth, and scalability, piezoelectric MUTs and capacitive MUTs are investigated.^24^ Because the sensitivity of both piezoelectric transducer and MUT is proportional to the area of the sensing element, they may suffer from poor sensitivity in compact PA systems. In recent years, several different optical ultrasound sensors have been explored in PA imaging.^19^^,^^25^^,^^26^ Their main advantages include wide bandwidth, low electromagnetic interference, and high sensitivity per unit area.^27^^–^^32^ These optical ultrasound sensors have undergone significant development, especially fiber laser sensor that has not been elaborated upon in detail in existing reviews. In this review, we first introduce the basic principles of different optical ultrasound sensors and their characteristics. Then we focus on the recent advances in optical ultrasound sensing for PA imaging and compare their imaging performance.

## 2 Optical Sensor Technology in PA Imaging

Various optical ultrasound sensors have been developed for PA imaging. According to the sensing principle, optical ultrasound sensors can be categorized into either resonance-based or nonresonance-based sensors.^29^^,^^31^ Resonance-based sensors operate by detecting variations in a resonance cavity caused by the acoustic pressure. These variations subsequently manifest as changes in the intensity, phase, or wavelength of the probing light. Examples of resonance-based ultrasound sensors include Fabry–Perot interferometer (FPI),^33^^,^^34^ fiber laser,^19^^,^^35^ Bragg grating,^36^^,^^37^ and whispering-gallery mode (WGM) microresonators.^38^^,^^39^ In comparison, nonresonance-based optical ultrasound sensors mainly utilize free-space methods or photoelastic approaches, such as Michelson interferometer,^40^^,^^41^ Mach–Zehnder interferometer (MZI),^42^^,^^43^ probe beam deflection technique,^44^^,^^45^ or laser Doppler sensors.^46^ In this review, we focus on the resonance-based optical ultrasound sensors.

### 2.1 Imaging Parameters of Optical Ultrasound Sensors

To compare the performance of ultrasound sensors in PA imaging, we use several parameters including, frequency, sensitivity, and acceptance angle.^28^^,^^31^ The working frequency of ultrasound sensors is critical for imaging quality. It contains two parameters, bandwidth and central frequency. Light-induced ultrasound waves possess a broad frequency spectrum, spanning from kHz to hundreds of MHz. Thus ultrasound sensors should ideally exhibit a wide bandwidth and high central frequency to enhance image resolution. If the impulse response of the ultrasound sensor has a Gaussian envelope, the axial resolution of PAM can be estimated by the formula, $R_{a}=0.88v_{a}/\Delta f$, where $v_{a}$ is the speed of sound and Δf is the bandwidth of ultrasound transducer.^47^^,^^48^ In addition, the axial resolution of PACT is also related to the bandwidth of ultrasound transducer.^49^ The bandwidth of resonance-based optical ultrasound sensors is dependent on the two concurrent processes during the detection, the optical resonance, and ultrasonic wave propagation.^50^ When the resonance mode is changed by the ultrasound wave, the resonance cavity need time to reach a steady state again. The spending time is comparable to the intracavity photon lifetime τ=Q/ω, where Q is the resonator quality factor and ω is the angular frequency of the light wave. So the frequency bandwidth can be limited by the quality factor Q.^51^ In addition, the bandwidth is influenced by the interaction between the ultrasound wave and the sensor, which is dependent on the fiber material and backing material, as well as their structure.^52^^–^^54^

Sensitivity is another key parameter in PA imaging. For typical resonance-based optical ultrasound sensors, the sensitivity S can be expressed as the change in transmission T induced by the acoustic pressure P, which can be expressed by $S=\frac{dT}{dP}=\frac{dT}{d\phi }\cdot \frac{d\phi }{dL_{o}}\cdot \frac{dL_{o}}{dP}$, where ϕ is the round-trip phase and $L_{o}$ is the optical path length of the resonator. The first term $\frac{dT}{d\phi }$ represents the maximal slope in the transmission spectral. The second term $\frac{d\phi }{dL_{o}}$ represents the phase change caused by the modulation of the cavity length. The last term $\frac{dL_{o}}{dP}$ represents the optical path length changed by the acoustic pressure, which is related with the mechanical and optomechanical properties of the sensor. The sensitivity can also be quantified by noise equivalent pressure (NEP) and noise equivalent pressure density (NEPD). NEP is defined as the minimum detectable signal pressure that equal to the noise amplitude.^18^^,^^28^ NEPD can show the sensitivity of sensors over the spectra. If the sensor sensitivity spectral density S(f) and noise amplitude spectral density of the sensor $N_{V}(f)$ are measured, NEPD can be calculated by $N(f)=N_{V}(f)/S(f)$.^55^

The acceptance angle is also crucial for PA imaging, which can measure the sensor’s ability to detect the ultrasound signal from different directions. Large acceptance angle is beneficial in reducing imaging artifact and improving the signal-to-noise ratio (SNR). An ultrasound sensor with a large acceptance angle can provide high imaging quality.^56^^–^^58^ Due to the varying distances from the sound source to different points on the sensor, the phase of the ultrasound wave reaching each point on the sensor differs. As the ultrasound signals with different phases received by the ultrasound sensor may cancel each other out, this results in a reduction in the signal. Consequently, smaller sensors often exhibit larger acceptance angles. In addition, the shape of the sensor has significant influence on the acceptance angle. For example, a ring-shaped sensor is superior to a disk-shaped one for near-field ultrasound detection because the ring shape minimizes the phase retardation.^27^ In addition, the acceptance angle of some sensors is tied on the detection principle, such as the fiber laser sensor. It utilizes the opposite refractive index changes in the perpendicular polarization modes. When the ultrasound wave is incident to the principal axis of the fiber laser sensor, the detected signal is the maximum. As the incident angle increases, the signal detected by the sensor gradually diminishes, reaching a minimum when the incident angle is 45 deg. This phenomenon occurs because, at this angle, the refractive index alterations in the perpendicular polarization modes are equivalent.^19^

### 2.2 Fabry–Perot Sensor

**Fig. 1:**
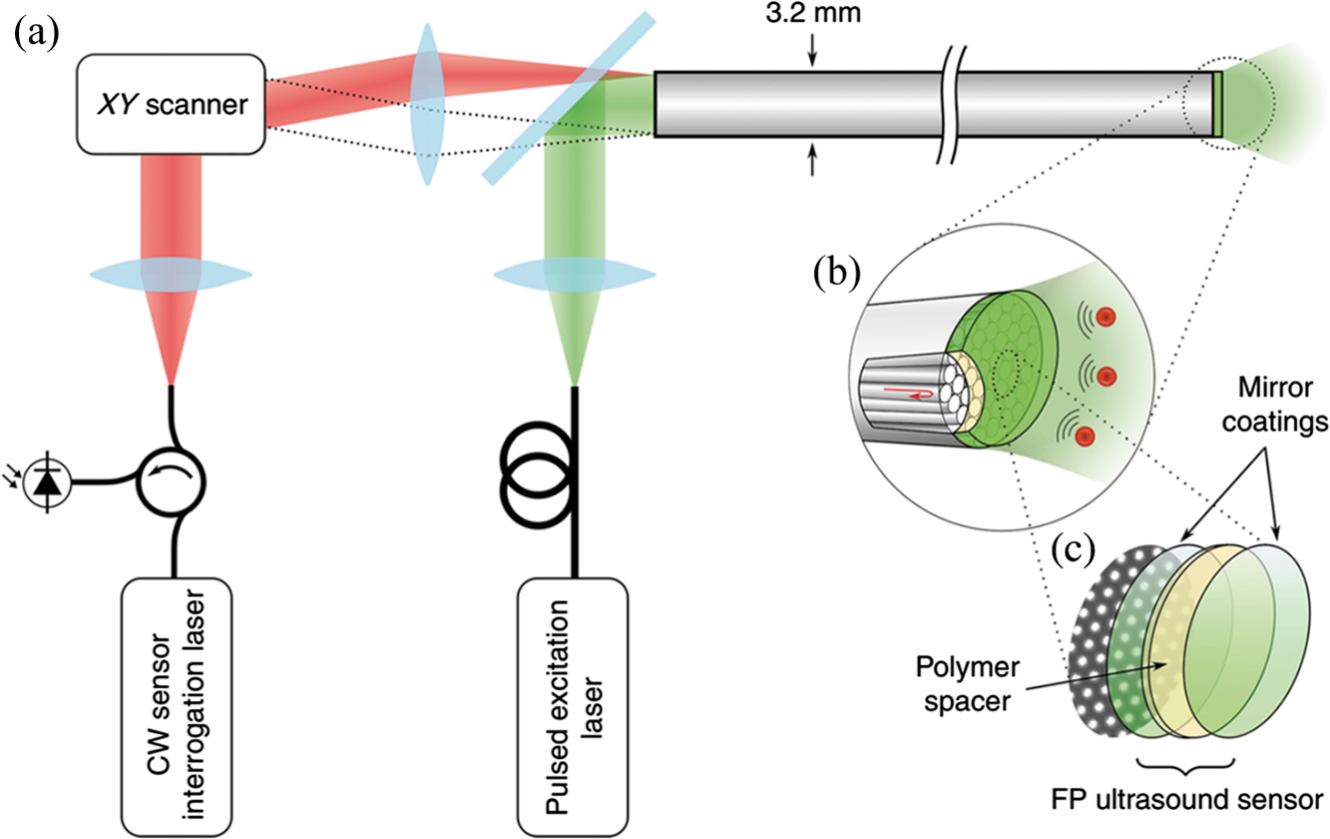
(a)–(c) PAE probe with planar FP sensor head. Reprinted with permission from Ref. 62, available under a CC-BY 4.0 license.

The FPI is composed of two highly reflective surfaces separated by a spacing material, together forming the FP cavity. As acoustic waves reach the surface, the optical thickness of the FPI shifts, causing a minor phase alteration and modulating the optical intensity.^52^^,^^59^ Zhang et al.^60^^,^^61^ introduced a planar FP ultrasound sensor that formed by a thin polymer (Parylene C) film spacer sandwiched between two dielectric dichroic mirrors. The system’s ability to provide PA images was demonstrated, but the imaging speed was slow for *in vivo* applications. Ansari et al.^33^^,^^62^ proposed a miniature forward-viewing 3D PA probe that comprises a coherent fiber bundle with an FP polymer-film ultrasound sensor at its distal end, as shown in Fig. 1. The optical fiber bundle with 50,000 cores acts as an ultrahigh-density ultrasound array, and the PA images can be obtained by sequentially scanning the input end of the bundle, which can greatly reduce the volume of the probe. The outer diameter of the probe is only 3.2 mm. However, the imaging speed of the system was limited by the pulse repetition frequency (PRF) of the excitation laser. The acquisition time of a figure can be more than 25 min. The imaging speed can be improved by increasing the excitation laser PRF and parallelizing the sensor read-out.^25^^,^^63^^,^^64^ Moreover, compressed sensing techniques can further accelerate imaging speeds.^65^^,^^66^

**Fig. 2:**
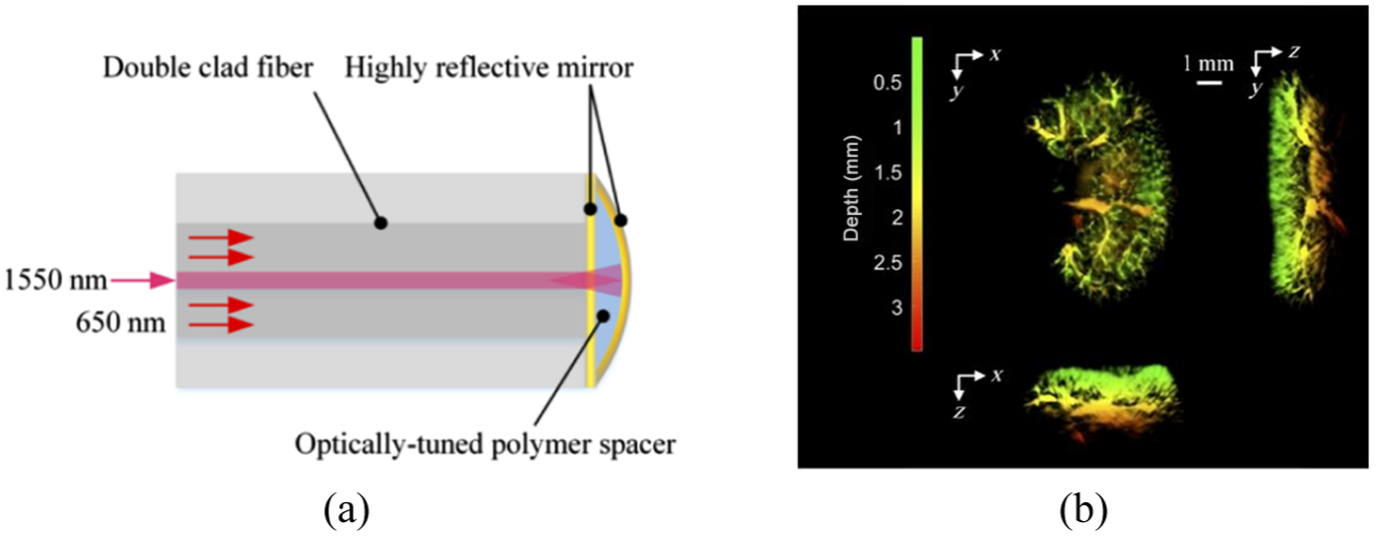
Plano-concave FP ultrasound sensor: (a) sensor structure and (b) the imaging results of an *ex vivo* mouse kidney. Reprinted with permission from Ref. 68, available under a CC-BY 4.0 license.

The performance of planar FP ultrasound sensor is limited by the beam walk-off and diffraction effects around the fiber-tip. To overcome these problems, a plano-concave FP ultrasound sensor structure is proposed.^67^ Guggenheim et al.^56^ proposed a high Q-factor plano-concave microresonator that has very high sensitivity with excellent broadband acoustic frequency response and wide directivity. This microresonator ultrasound sensor has much higher Q-factor ($>10^{5}$) than planar FP ultrasound sensor because the plano-concave structure can precisely correct for the divergence by refocusing the light upon each round trip and preventing the beam from walking off laterally. Thus the sensitivity of the microresonator is greatly improved, and the NEP can be lower than $1.6\;\mathrm{mPa}/{\mathrm{Hz}}^{1/2}$. Chen et al.^68^ proposed photothermally tunable FP sensor for PA mesoscopy, as shown in Fig. 2(a). The NEPD of the FP sensor is $40\;\mathrm{mPa}/{\mathrm{Hz}}^{1/2}$ with an acoustic detection bandwidth up to 30 MHz. The PA image of an *ex vivo* mouse kidney reconstructed by the FP sensor is shown in Fig. 2(b).

**Fig. 3:**
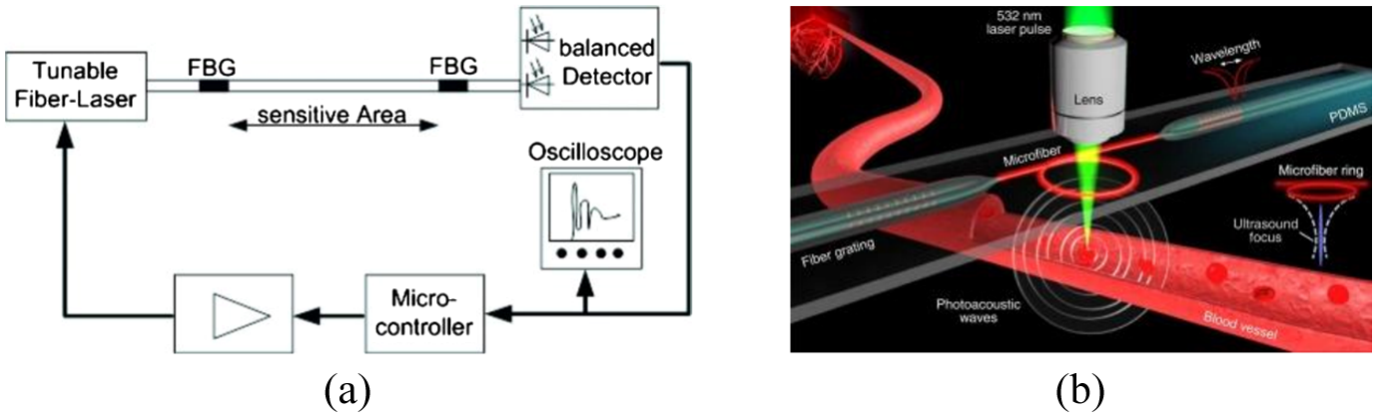
FBGs-based FP ultrasound sensor system. (a) FP interferometer formed by two FBGs. Reprinted with permission from Ref. 69. (b) Schematic of the transparent microfiber ultrasound sensor for PA imaging. Reprinted with permission from Ref. 70, available under a CC-BY 4.0 license.

Since fiber Bragg grating (FBG) can be used as mirrors in fiber-based interferometers, several researchers proposed FP ultrasound detector based on FBG technology. For FP interferometer that formed by FBGs, the sensing area is the region between the two FBGs, as shown in Fig. 3(a). By measuring variations of the refraction index (RI) induced by the acoustic pressure, the acoustic signal can be detected.^71^ Gruen et al.^69^ realized an integrating line detector with a fiber-based FP interferometer formed by two FBGs. The reflectivity of an FBG is 81%, and the distance between the FBGs is 11.5 cm. The team experimented with bristle knots and ants using FP glass-fiber interferometers. Wang et al.^72^ proposed a microfiber FBG-based FP interferometric acoustic transducer and verified its performance by the imaging studies of human hairs. Ma et al.^70^ proposed a Fabry–Perot ultrasound sensor that formed by a microfiber loop sandwiched by a pair of inline Bragg gratings, as shown in Fig. 3(b). Although constrained by imaging sensitivity, the needle-like focus of the microfiber serves to mitigate the degradation of both resolution and signal amplitude in out-of-focus regions.

### 2.3 Fiber Laser Sensor

A fiber laser is a type of laser where the active gain medium is an optical fiber infused with rare-earth elements such as erbium, ytterbium, and neodymium. When equipped with two reflective mirrors, a fiber laser configuration is established. A specific type of short-cavity fiber laser, known as either a distributed feedback or distributed Bragg reflector laser, is adept at detecting ultraweak signals, including strains and acoustic waves. Furthermore, the application of the wavelength-division multiplexing technique allows these fiber laser sensors to be integrated into a sensor array.^73^

**Fig. 4:**
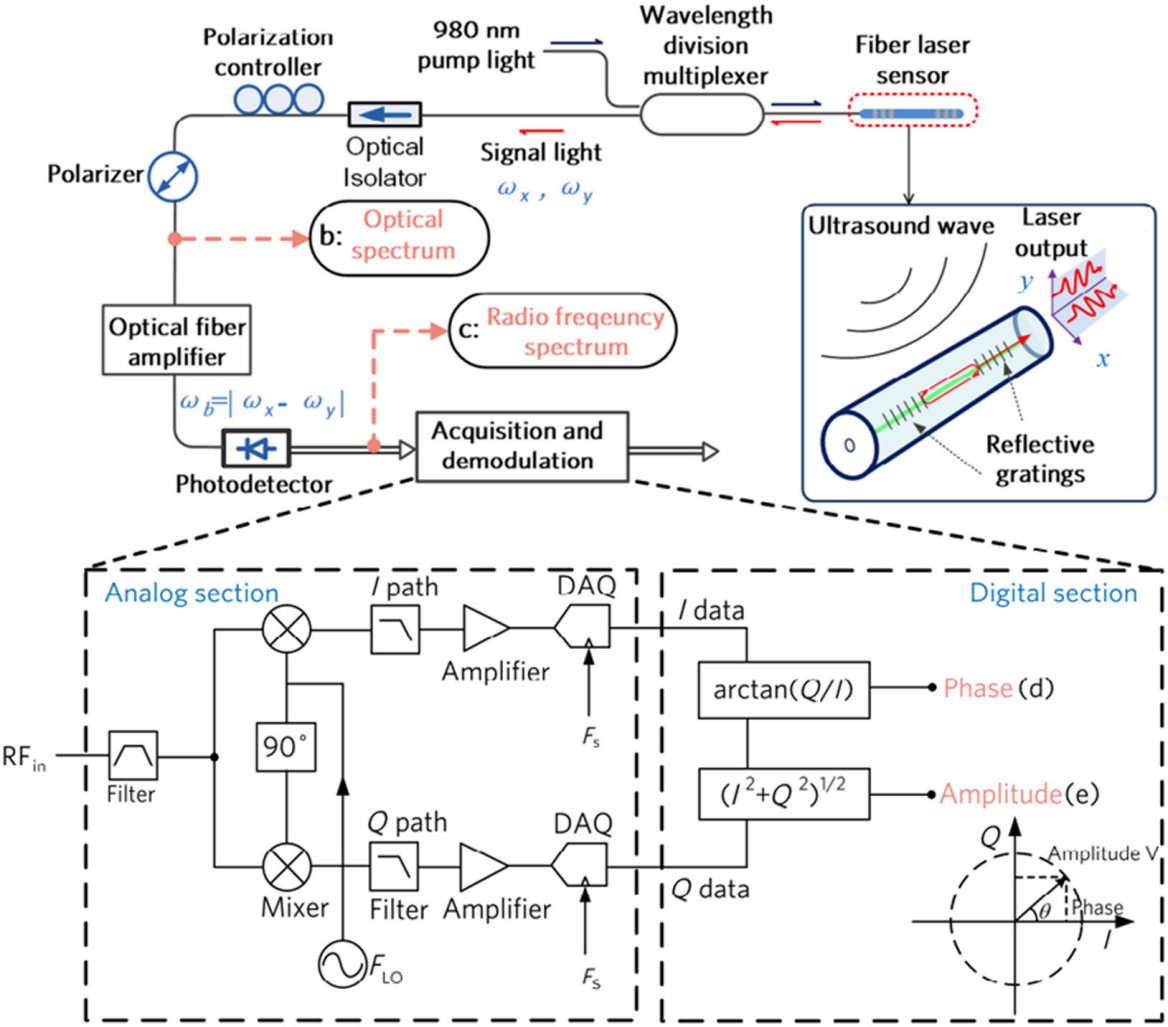
Fiber laser ultrasonic sensing system. Reprinted with permission from Ref. 19, available under a CC-BY 4.0 license.

Liang et al.^19^ introduced a fiber laser sensor to detect high-frequency ultrasound waves and the ultrasonic sensing system is shown in Fig. 4. This sensor amplifies the acoustic response based on the frequency change of the signal light to measure ultrasound, specifically by gauging the acoustically induced optical phase change. The NEPD of this system is below $1.5\;\mathrm{mPa}/{\mathrm{Hz}}^{1/2}$ within a measured frequency range of 5 to 25 MHz. The optical phase detection uses the beating signal between two different-polarized laser beams, offering resilience against thermal drift and vibrational disturbances.

Guan et al.^35^^,^^74^^,^^75^ proposed a sensor with a sensitive primitive consisting of a fiber laser. This laser is constructed by inscribing two wavelength-matched FBGs into an Er-Yb co-doped fiber, as depicted in Fig. 4. These gratings exhibit a strong reflection at ∼1550  nm, which facilitates the generation of laser light. Notably, this wavelength corresponds to the peak luminescence efficiency of the fiber gain ion, enabling the laser to achieve a significant gain. The gratings have a length ranging from 2 to 6 mm, and their spacing can vary between 0.5 and 10 mm. Emitting laser light from cavities shorter than 2 mm is challenging due to the restricted doping concentration. The sensor leverages the inherent birefringence of the optical fiber. The fiber’s natural processing makes it weakly birefringent, leading to the generation of two laser beams with distinct frequencies on the x and y polarization axes. The two orthogonal modes produce a beat signal in a detectable GHz frequency range. When ultrasonic waves induce vibrations in the optical fiber, the refractive index changes in the x- and y-polarized modes are equal but opposite. This phenomenon is utilized in the optical heterodyne detection method. Both modes respond similar to low-frequency disturbances, such as thermal and mechanical vibrations, which can be minimized in the beat signal.

In the fiber laser ultrasonic sensing system, an erbium-doped fiber amplifier is used to boost the light power, allowing the photodetector to function in the shot-noise-limited region, further improving the SNR. Finally, the photodetector transforms the optical signal into a radio frequency signal.

Ultrasonic sensing systems primarily experience two types of noise: phase noise and intensity noise.^76^ In fiber laser PA imaging systems, the predominant sources of phase noise include the inherent noise from the fiber laser (a sensitive element), spontaneous radiation noise from the fiber amplifier, thermal and scattering noise from the photodetector, and noise from the data acquisition (DAQ) system. The total system noise is primarily attributed to the fiber laser, optical amplifier, and DAQ system. At a frequency of 3 MHz, when the noise from the DAQ system is approximately −140  dBc/Hz, the system’s noise power density stands at about −130  dBc/Hz. The sensing system shown in Fig. 4 remains unaffected by intensity fluctuations and the coupling efficiency between intensity and phase noise is minimal, ranging from 1% to 3%. Consequently, the acoustic sensitivity is solely limited by the phase noise.

**Fig. 5:**
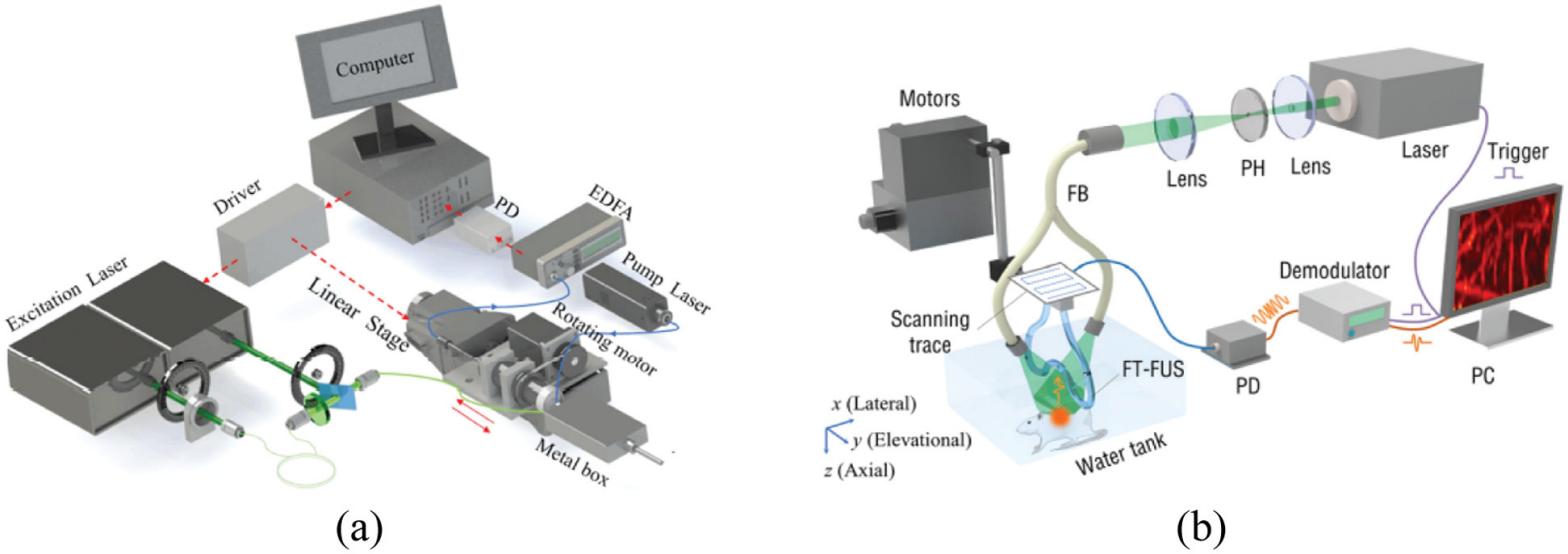
Fiber laser PA system: (a) PAE system, reprinted with permission from Ref. 19, available under a CC-BY 4.0 license. (b) PACT system, reprinted with permission from Ref. 79 with permission.

Liang et al.^19^^,^^77^ and Zhou et al.^78^ applied the fiber laser to the PAM and PAE. The PAE system consists of a PA probe, a dual-wavelength laser source, a sensor interrogation unit, mechanical scanners, and DAQ and control modules [Fig. 5(a)]. In addition, Bai et al.^79^ applied the sensor to the PACT [Fig. 5(b)]. The imaging depth can be tuned by bending the fiber laser sensor into different curvatures using the customized holder.

### 2.4 π-Phase-Shifted FBG

**Fig. 6:**
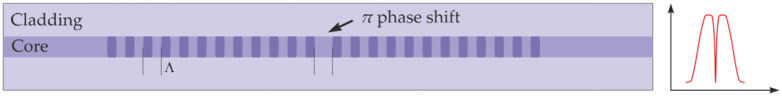
Schematic and reflection spectrum of a π-FBG. Λ is the grating pitch. Reprinted with permission from Ref. 80, available under a CC-BY 4.0 license.

Another FBG-based sensor, π-phase-shift FBG (π-FBG) is an alternative optical sensor that used in PA imaging. Bragg grating is a transparent structure with a periodic variation of refractive index. π-FBG contains a phase jump of π at the center of the FBG, forming a region analogous to the cavity of FPI [Fig. 6(a)].^80^^–^^82^ This phase jump leads to a narrow spectral notch at the center of the reflection bandwidth of the grating, allowing highly sensitive ultrasonic detection.^83^^–^^85^

**Fig. 7:**
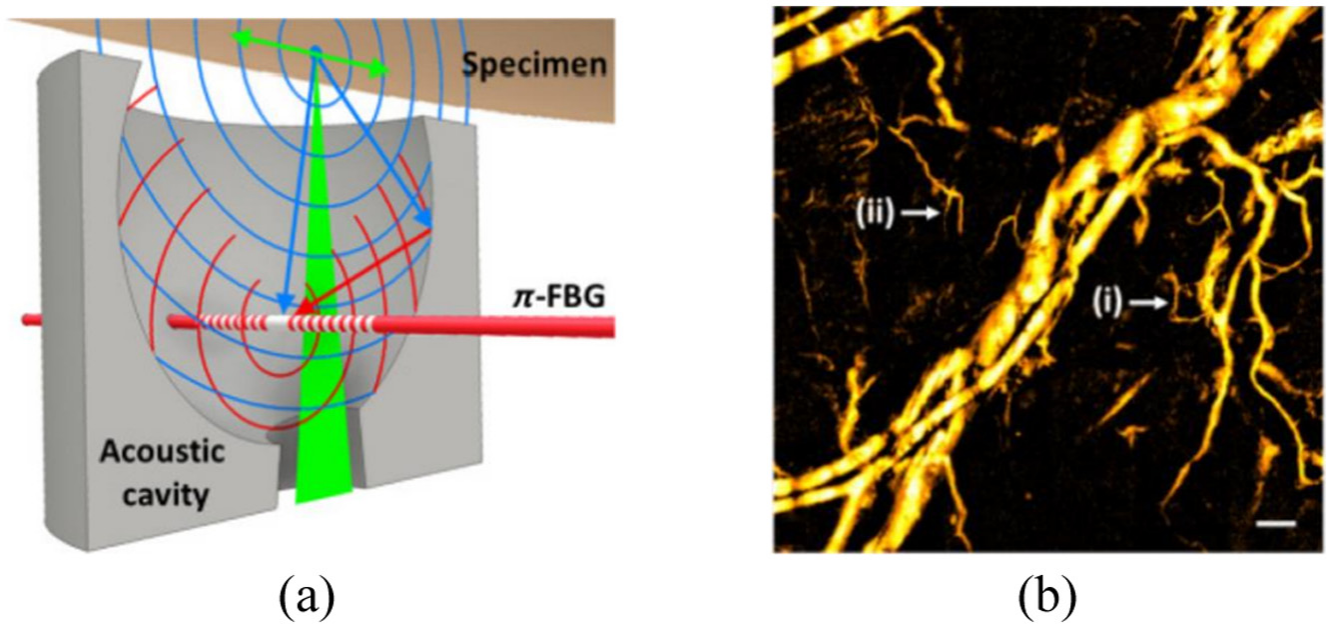
π-FBG PA microscope: (a) schematic of the π-FBG-based sensor and (b) the imaging results of a mouse ear *in vivo*. Reprinted with permission from Ref. 88, available under a CC-BY 4.0 license.

The imaging systems based on π-FBG have been successfully applied in PA imaging. Rosenthal et al.^86^ proposed an intravascular PA catheter with a diameter of 1 mm that consisted of a fiber with a π-FBG written close to its tip and an additional illuminating fiber. The NEP of the π-FBG over 16 MHz bandwidth was found to be 100 Pa. The narrow transmission spectrum of π-FBG was utilized to reduce the amplified spontaneous emission noise and improve the sensitivity. To translate the ultrasound signal into intensity shifts, the fiber-based Mach– MZI was used for active demodulation. A healthy stented artery *ex vivo* was imaged by the catheter and the results showed that the system had great stability even strong vibrations were applied to the catheter. Wissmeyer et al.^87^ presented an all-optical PA microscope and its biological imaging results. The adopted π-FBG had a narrow resonance width of 8 pm at −3  dB and two distinct frequency bands at −6  dB, ranging from 7 to 27 MHz and from 62 to 77 MHz, which contributed to obtain the high-resolution PA images. Shnaiderman et al.^88^ described a miniaturized PA sensor with π-FBG embedded in an acoustic cavity, as shown in Fig. 7(a). Though the Q factor of the sensor is moderate, the sensitivity could be compensated by acoustic cavity signal amplification, achieving the NEP of 88 Pa. A mouse ear imaged by the sensor *in vivo* is shown in Fig. 7(b).

**Fig. 8:**
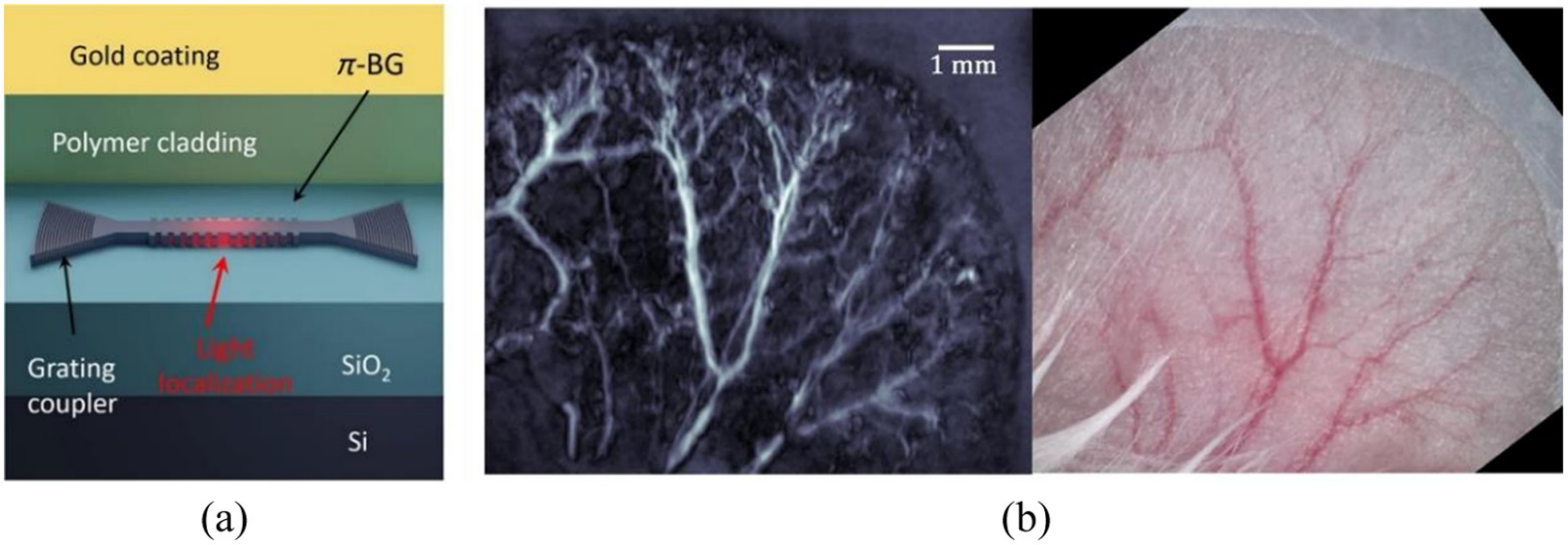
SPADE PA microtomography: (a) illustration of the silicon-photonics layer structure and (b) the imaging results of a mouse ear *in vivo*. Reprinted with permission from Ref. 37, available under a CC-BY 4.0 license.

To further reduce the sensor dimensions and achieve a higher center frequency, fiber-optic waveguide has been applied to fabricate the π-FBG sensor. Rosenthal et al.^89^ had demonstrated a miniaturized wideband ultrasound sensor based on π-phase shifted waveguide Bragg grating (π-WBG) that embedded in a silicon-on-insulator (SOI) photonic platform. Shnaiderman et al.^36^ developed a point-like silicon waveguide–etalon detector (SWED) with a sensing area of only 220  nm×500  nm using SOI technology. The point-like SWED reached an ultrawide bandwidth of 230 MHz at −6  dB and could provide super-resolution detection and imaging performance. In addition, the small size of SWED provides a potential method to build very dense ultrasound arrays on a silicon chip. Hazan et al.^37^ proposed a miniaturized silicon-photonics acoustic detector (SPADE) with NEPs down to $2.2\;\mathrm{mPa}\;{\mathrm{Hz}}^{-1/2}$ and a bandwidth above 200 MHz that capable of tomographic imaging. The π-WBG in SOI that coated with the elastomer polydimethylsiloxane, which can enhance the sensitivity and reduce the parasitic effect of surface acoustic waves. The imaging performance of SPADE was tested by both dark knot *ex vivo* and mouse ear *in vivo*, as shown in Fig. 8(b).

### 2.5 Whispering Gallery Mode

In optical WGM, total internal reflection can confine light waves within a closed circular microcavity. The resonance is achieved when the optical path length matches an integer multiple of the laser’s wavelength.^90^^,^^91^ This section delves into ultrasound transducers featuring various resonant microcavity shapes, such as microrings, microspheres, and microbubbles.

**Fig. 9:**
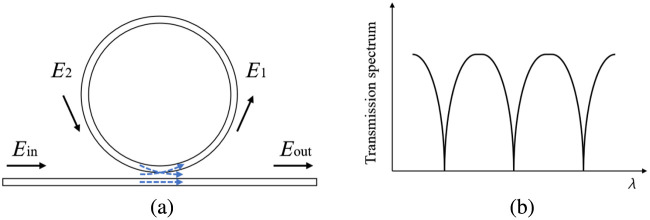
Microring resonator sensor: (a) geometry and (b) typical transmission spectrum.

The microring resonator (MRR) is composed of a bus waveguide and a circular waveguide, depicted in Fig. 9(a). A laser is introduced from one end of the bus and is evanescently coupled to the ring waveguide through a low-dielectric gap separating the bus from the circular waveguide. When the circumference of the circular waveguide corresponds to integer multiples of the laser’s wavelength, resonance is achieved, resulting in distinct dips in the transmission spectrum, as illustrated in Fig. 9(b).^92^^,^^93^ Ultrasound waves can deform the MRR, altering the effective RI of the guided mode due to the elasto-optic effect. This causes a shift in the resonance wavelength, allowing the detection of ultrasound waves by monitoring the modulated output intensity.

The MRR’s submicron thickness induces an acoustomechanical resonance within the gigahertz range, enabling a uniform frequency response from DC up to several hundred megahertz.^94^ This ultrasound sensor stands out due to its high Q factor and pronounced resonance from multibeam interference, offering both exceptional sensitivity and a broad bandwidth. Zhang et al.^95^ introduced a polystyrene (PS) microring sensor. This sensor is characterized by a ring and bus structure with dimensions of 60  μm in diameter and 1.4  μm in height. The sensor’s resonance bandwidth is 6 pm with Q factor of $1.3\times 10^{5}$. Furthermore, it has a bandwidth of 350 MHz and a NEP of 105 Pa within this range.

**Fig. 10:**
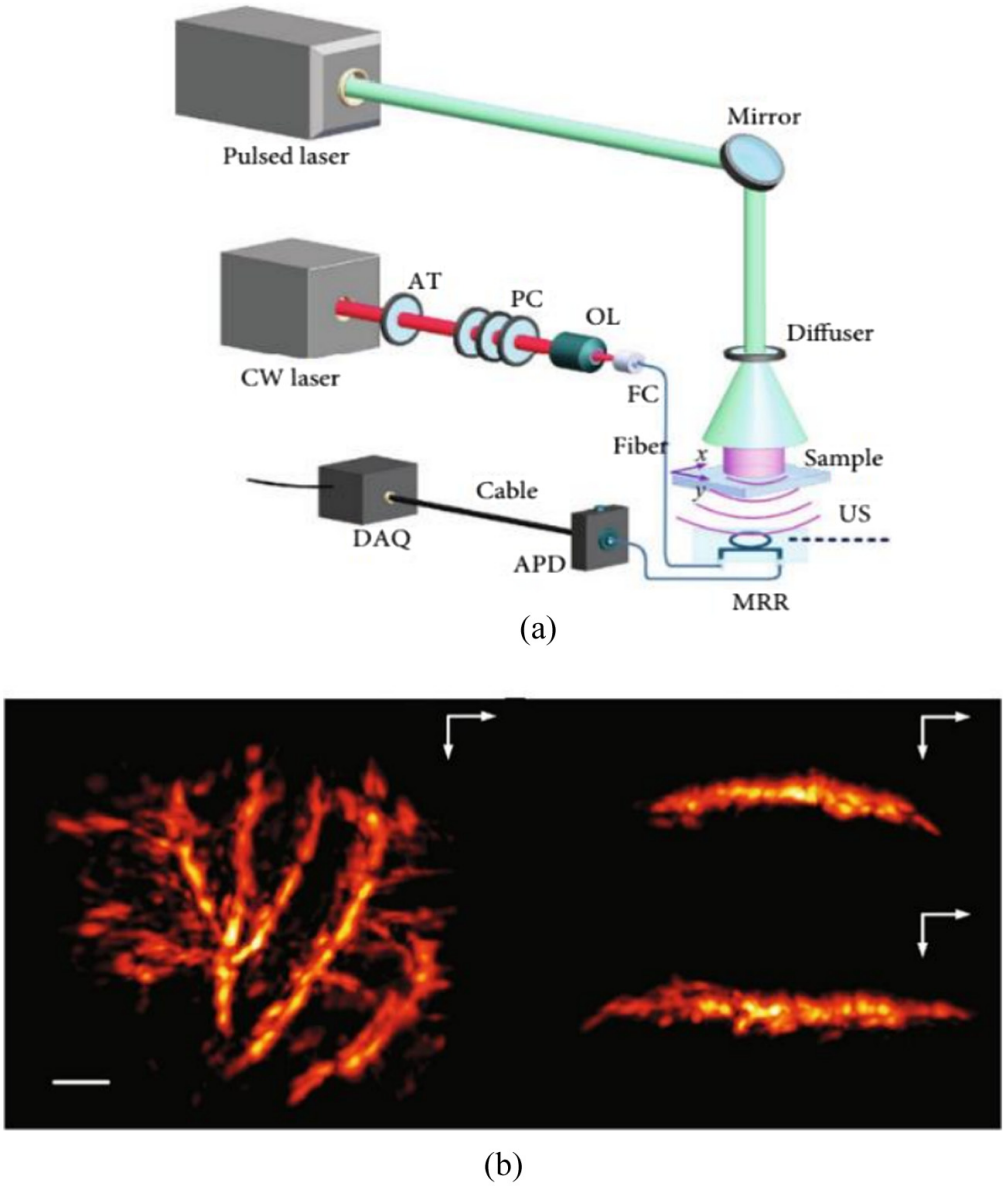
MRR 3D-PACT: (a) schematic of the imaging system and (b) the imaging results. Reprinted with permission from Ref. 94, available under a CC-BY 4.0 license.

Encasing MRR sensors in an acoustic impedance-matched protective layer enhances their reliability and stability, making them more suitable for *in vivo* applications. Rong et al.^94^ introduced an MRR sensor with an 80-μm diameter, crafted using nanoimprint lithography. This sensor has a Q factor of $4.6\times 10^{4}$ and a NEP of 81 Pa, complemented by its ∼23  MHz detection bandwidth and a 90 deg acceptance angle. Building on this, Rong et al. developed a 3D-PACT system centered around this MRR sensor, as shown in Fig. 10(a). This system employs a low RI polymer known for its biocompatibility. The sample undergoes scanning on a motorized three-axis stage, with a narrowband tunable laser serving as the detection light source. This system can provide lateral and axial resolutions of ∼114 and ∼57  μm. The system was validated by imaging human hair, leaf veins, isolated mouse brains as well as *in vivo* mouse ears and tadpoles. The results showed that the system can obtain high SNR and high-contrast 3D PA images. The PA images of the mouse ear that reconstructed by this system are shown in Fig. 10(b).

**Fig. 11:**
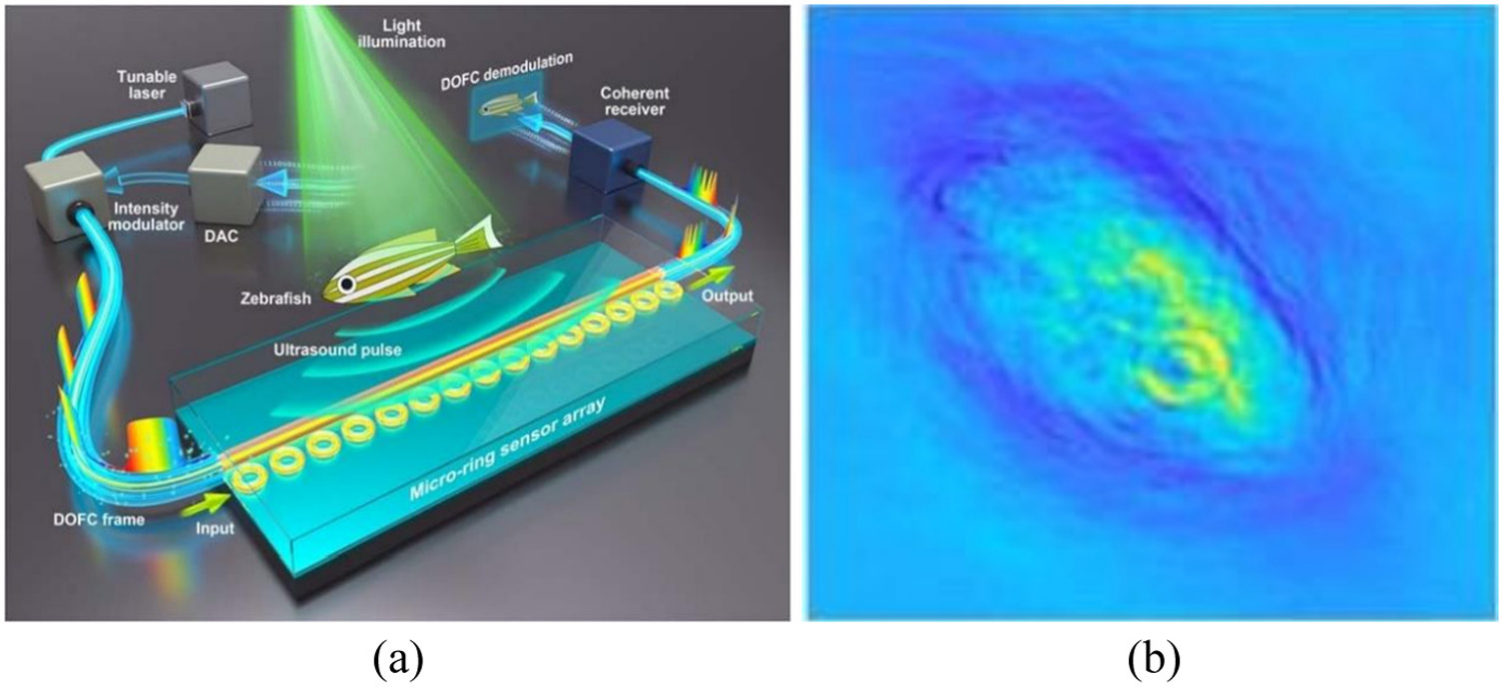
MRR array PACT: (a) schematic of the imaging system and (b) the imaging result of a 3-month-old adult zebrafish. Reprinted with permission from Ref. 96, available under a CC-BY 4.0 license.

Due to the ability of digital optical frequency comb (DOFC) to generate ultranarrow and tunable combs, it can be used to locate the resonance frequency of an array of microring sensors in parallel with high precision, enabling a one-off measurement of the transmission spectrum using only a single photoreceiver, which simplifies the use of PACT systems with arrays of microrings. Pan et al.^96^ proposed a PACT system based on an array of 15 microring sensors, as shown in Fig. 11. The chalcogenide-based MRR sensors have high Q factors ranging from $5\times 10^{5}$ to $7\times 10^{5}$, while each element has a bandwidth of 175 MHz at −6  dB, a NEP of $2.2\;\mathrm{mPa}\;{\mathrm{Hz}}^{-1/2}$, and an acceptance angle of ±30  deg. These sensors were tuned to slightly different resonant frequencies, and by DOFC, Pan et al. implemented a PACT system and scanned leaf veins, zebrafish at different growth stages.

**Fig. 12:**
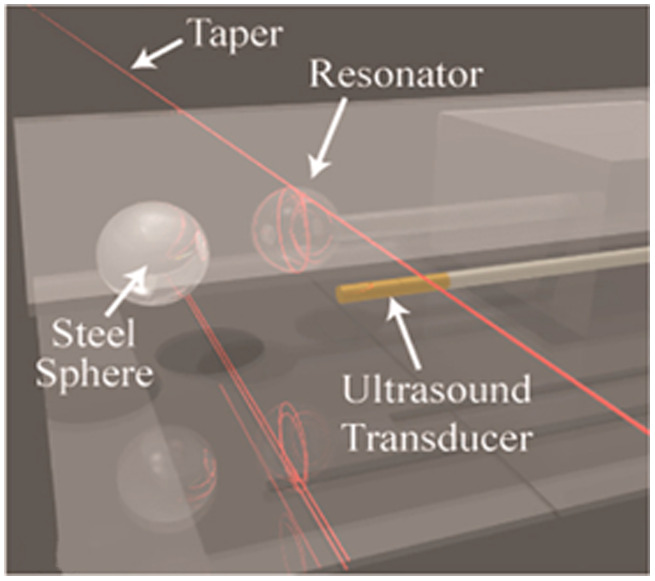
Sensing system of microsphere resonator sensor. Reprinted with permission from Ref. 97, available under a CC-BY 4.0 license.

The microsphere resonator sensor consists of a taper fiber and a microsphere cavity, as shown in Fig. 12.^97^ Ultrasound deforms the microsphere cavity and causes changes in the RI of surrounding medium and spheres at the same time, which affects the coupling mode.^98^ Sun et al.^38^ proposed an method to fabricate microsphere sensors and developed two types of encapsulated microsphere resonators with different cavity materials. The silica microsphere sensor has a Q factor of $∼10^{6}$ and 160 Pa NEP at 20 MHz, whereas the PS microsphere sensor has a Q factor of $∼10^{5}$ and 100 Pa NEP at 20 MHz. Sun et al. applied the microsphere sensor to PAM with a lateral resolution of ∼5  μm and successfully imaged hairs and leaf veins in 3D.

**Fig. 13:**
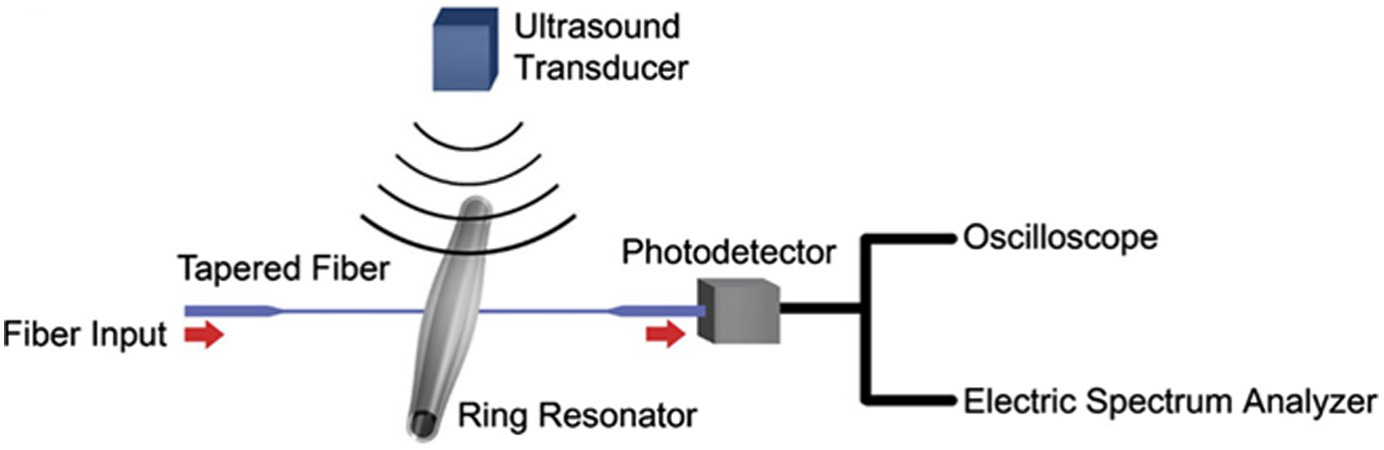
Schematic of the experimental setup for pressure wave detection using MBR sensor. Reprinted with permission from Ref. 99, available under a CC-BY 4.0 license.

Microbubble resonators (MBRs) are manufactured using hollow capillary tubes, which have greater deformation compared to microrings and microspheres. It can have a Q factor of $10^{7}$, and the schematic of the sensing system with MBR sensor is shown in Fig. 13.^99^ Tu et al.^100^ proposed an packaged optical MBRs sensor with a broad bandwidth (10 Hz to 100 kHz). This size of the sensor is 140  μm in diameter with a wall thickness of 5  μm. It has a Q factor of $5.2\times 10^{5}$ and a NEP of $2.2\;\mathrm{mPa}/{\mathrm{Hz}}^{1/2}$. Tu et al. applied the sensor to underwater acoustic wave detection. They recorded responses in two orthogonal directions, spanning angular ranges of 75.6 deg and 105.5 deg (at −6  dB). MBRs have been successfully applied to PA sensing and have the potential to be used for PA imaging.

### 2.6 Summary of Resonance-Based Ultrasound Sensors

The primary characteristics of resonance-based ultrasound sensors are detailed in Table 1. These ultrasound sensors with optical resonance are small but have high sensitivity and broad bandwidth. FP ultrasound sensors are commonly used for ultrasound detection. They can achieve high Q factor, have low NEP, and large acceptance angle, empowering them to capture PA images with superior resolution and contrast. The FP ultrasound sensor can be fabricated at the tip of optical bundle and acting as a high-density ultrasound array to realize 3D forward-viewing PA imaging. However, FP ultrasound sensors require locking the probe laser wavelength to the resonance frequency of the FP cavity. As for fiber laser sensor, drawing on the birefringence principle, it adopts heterodyne phase detection for detecting ultrasound waves, which does not require an additional laser to scan and is insensitive to perturbations, such as temperature and optical intensity change. As a result, the fiber laser sensor can be sensitive and stable. But the fiber laser sensor has directivity, it should be rotated to the most sensitive angle before measuring. π-FBG sensors are increasingly used in PA imaging due to their broad bandwidth and high sensitivity. Based on SOI technology, π-FBG can have compact configuration for high-density arrays. However, akin to FP sensors, π-FBG sensors also need use additional laser for detection. WGM sensors can achieve the highest Q factors and have ultrabroad bandwidth and wide angular response. The small size and transparent WGM sensors can be conveniently integrated into OR-PAM with high-NA objective lens that has a limited working distance.^50^ Yet current fabrication techniques for WGM remain challenging.

**Table 1:** Performance of optical ultrasound sensors.

Type	Size (mm)	Sensitivity	Resolution (lateral/axial) (μm)	Bandwidth at −3 dB (MHz)	Acceptant angle (deg)	Reference
FP	Outer diameter: 3.2	0.5 to 1.26 kPa	45 to 170/31	34	—	62
FP	Thicknesses: 0.03 to 0.53	2.6 Pa (minimum)	20/36	40	180	56
FP	Outer diameter: 1.5	∼700 Pa	OR-PAM: 3/-AR-PAM: ∼320/∼210	10.2	—	70
Fiber laser	Probe size: 2	$1.5\;\mathrm{mPa}{\;\mathrm{Hz}}^{-1/2}$	7.4/—	20	60	19
Fiber laser	Curvature radius: 25	25 Pa	150/85	20	60	79
π-FBG	220 nm×500 nm	$9\;\mathrm{mPa}\;{\mathrm{Hz}}^{-1/2}$	0.65/—	230 (−6 dB)	148	36
π-FBG	Diameter: 1.3	108 Pa	124/18.6	40.4 (−6 dB)		54
Microring	Diameter: 60 μm	105 Pa	—/<3	350	—	95
Microring	Diameter: 80 μm	81 Pa	114/57	23	90	94
Microring	Diameter: 40 μm	7.1 Pa	50.4/43.6	175 (−6 dB)	60	96
Microsphere	Diameter: 20 μm	160 Pa	5/—	70 (−6 dB)	—	38

## 3 Conclusion

Optical ultrasound sensors offer distinct advantages in biomedical imaging, including heightened sensitivity, flexibility, and compactness when juxtaposed with traditional ultrasound transducers. In this review, the commonly used optical ultrasound sensors are introduced, such as FP, fiber laser, Bragg grating, and WGM. The principle and characteristics of these optical ultrasound sensors are presented in detail. Both their merits and potential drawbacks are discussed. FP ultrasound sensors and π-FBG sensors shine with their broad bandwidth and superior sensitivity but necessitate an additional tunable narrow line-width continuous wave laser. The fiber laser sensors use heterodyne phase detection, so they are insensitive to perturbations, such as optical intensity change. But the fiber laser sensors are more sensitive to the direction angle. WGM sensors can achieve higher sensitivities because of the higher Q factors but have difficult fabrication process.

Optical ultrasound sensors herald novel opportunities in PA imaging, notably in the realm of PAE. As silicon photonics technology continues to advance, the potential for optical ultrasound sensors in parallel sensing is poised to expand. The future holds promise for optical ultrasound sensors that combine small size, heightened sensitivity, expansive bandwidth, and parallel detection, paving the way for a broader spectrum of applications in biomedical imaging.

## Data Availability

Data sharing is not applicable to this article, as no new data were created or analyzed.
